# RBLOSUM performs better than CorBLOSUM with lesser error per query

**DOI:** 10.1186/s13104-018-3415-5

**Published:** 2018-05-21

**Authors:** Renganayaki Govindarajan, Biji Christopher Leela, Achuthsankar S. Nair

**Affiliations:** Department of Computational Biology and Bioinformatics, University of Kerala, Thiruvananthapuram, Kerala India

**Keywords:** Substitution matrix, Sequence similarity search, BLOSUM, RBLOSUM, CorBLOSUM

## Abstract

**Objective:**

BLOSUM matrices serve as standard matrices for many protein sequence alignment programs. BLOSUM matrices have been constructed using BLOCKS version_5.0_ with 27,102 BLOCKS, whereas the latest updated version_14.3_ has 6,739,916 BLOCKS. We read with interest the research article by Hess et al. (BMC Bioinform 17:189, 2016) on CorBLOSUM, wherein it is argued that an inaccuracy in the BLOSUM code affects the cluster memberships of sequences. They show that replacing the integer based clustering threshold to floating point arguably improves the performances of CorBLOSUM over BLOSUM and RBLOSUM matrices. They compare BLOSUM62_14.3_ against RBLOSUM69, with relative entropies of 0.2685 and 0.2662 respectively. The present work attempts to repeat the computation to verify the respective analog matrices.

**Results:**

In our attempt to repeat the computation, we observed that the relative entropy of BLOSUM62_14.3_ is 0.2360 and BLOSUM50_14.3_ is 0.1198. As only matrices of similar entropies can be compared, BLOSUM62 can be compared only with RBLOSUM66 and BLOSUM50 can be compared only with RBLOSUM56. We conducted experiments with Astral data sets, and demonstrated the improved accuracy in the coverage. Our results imply that RBLOSUM performs statistically better than CorBLOSUM and BLOSUM matrices.

**Electronic supplementary material:**

The online version of this article (10.1186/s13104-018-3415-5) contains supplementary material, which is available to authorized users.

## Introduction

Sequence alignment is at the center stage of bioinformatics and amino acid substitution matrices play a major role in sequence alignment and homologous search. Alignment also serves as an initial method for de novo secondary structure prediction of proteins as well as knowledge based structure prediction. BLOSUM matrices [1] were developed more than two decades ago and were empirically derived from BLOCKS [2] database version 5.0. BLOSUM50 and BLOSUM62 are the two widely used matrices in all alignment programs [3]. With the increasing accumulation of sequences in public databases, the BLOCKS database has also been updated. The latest release of BLOCKS database is version 14.3 comprising of *6,739,916* sequences. How well the BLOSUM matrix (computed in 1992) is effective in faithfully representing the available data is a significant question. New set of sequences with varying amino acid composition were introduced in different clusters of BLOCKS database, that would be either under-represented or over-represented in the BLOCKSv5 database. Thus the increase in protein conserved regions in the BLOCKS database helps in deriving improved scoring matrices (see Additional file 1). Development and improvement of scoring matrices are crucial for identifying and aligning more distant homologs in similarity studies (see Additional file 2). Improvement in scoring matrices tends to increase the statistical significance and accuracy of alignments. Many studies have been undertaken on BLOSUM matrices, related to recalculating clustering steps [4] and parametrization [5]. These studies are seen carried out using the initial BLOCKS release version5.0. Hess et al. have recently proposed CorBLOSUM [6] by addressing an inaccuracy in the BLOSUM code. They relied on computing the matrices by changing the datatype threshold from integer to float. They claim that CorBLOSUM is performing better than BLOSUM and RBLOSUM. The expansion of BLOCKS database prompted us to recompute BLOSUM and also RBLOSUM matrices, using the latest version of the BLOCKS database.

## Main text

### Methods

Different BLOSUM matrix variants were created using BLOCKS version5, version13+ and version14.3 which were obtained from http://blocks.fhcrc.blocks/uploads/blocks.tar.gz. The BLOSUM matrices for the updated blocks were recomputed using the Henikoff and Henikoff [1] algorithm and the source code has been obtained from ftp://ftp.ncbi.nih.gov/repository/blocks/unix/blosum/programs/. Similarly RBLOSUM matrices were recomputed using the algorithm developed by Styczynski et al. [4] and the respective programs were obtained from http://web.mit.edu/bamel/blosum/revised_blosum.c. CorBLOSUM [6] matrices were directly obtained from http://www.cbs.tu-darmstadt.de/CorBLOSUM/. BLOSUM50 and BLOSUM62, the most widely used BLOSUM matrices, were considered in the present study. In order to find the analog matrices in RBLOSUM, matrix computing algorithms were executed for various percentage identity thresholds from 45 to 70. The RBLOSUM matrix with relative entropy closest to the BLOSUM matrices [4] were identified for BLOCKS release version5, version13+ and version14.3.

### Database

To evaluate the performance of each matrix, ASTRAL [7–9] database was used. ASTRAL is a benchmark data set, created based on SCOP, which classifies proteins into a hierarchical structure of classes, folds, superfamilies, and families based on their structure and functionality [10, 11]. In ASTRAL40 subset, sequences with more than 40% identity were eliminated and remaining were used for further study. This subset aids in identifying the ability of the substitution matrix to detect remote homologs. This non-redundant set numbering 13761 was obtained from http://scop.berkeley.edu/downloads/scopeseq-2.06/astral-scopedom-seqres-gd-sel-gs-bib-40-2.06.fa.

The data set was classified into training and test data set, based on folds. The training set consisted of 629-fold, 1004 super families and 7238 sequences, whereas the test set consisted of 626-fold, 1002 super families and 6522 sequences. Homologous search was performed on the training set with different gap opening and extension penalty. Gap parameter giving highest coverage was used to evaluate the test set. 20,95,25,616 pairwise alignments were performed using the training set and it was evaluated for further study.

### Search methods

In order to evaluate the effectiveness of the matrices computed based on the Astral subset, homologous search study was conducted using Smith-Waterman [12] local alignment algorithm. We used the SSEARCH implementation of the Smith-Waterman algorithm by Pearson. SSEARCH [13] has been shown to possess higher accuracy than BLAST in assessing the performance of different substitution matrices [14]. In addition to gap penalties, other parameters were set to default values for conducting similarity search. Previous works [15] have shown that optimizing gap penalties will boost the performance [16]. These penalties correspond to commonly used parameter settings in homology search tools such as BLAST [17] and SSEARCH [13]. Homologous search was performed for each combination of matrix, gap open and gap extension penalties. The best performing gap parameter for each matrix, on each of the tested ASTRAL database, was further studied.

### Performance evaluation

Pairwise sequence comparison and evaluation method (PSCE) developed by Price et al. [18] was used to evaluate the statistical significance of the substitution matrices, using the Bayesian bootstrap. Bayesian bootstraping is a resampling procedure which is operationally similar to the standard non-parametric bootstrap [19]. Sequences were assigned varying weights drawn from a Dirichlet distribution, in the case of Bayesian bootstraping [18]. PSCE uses coverage vs. errors per query (CVE) as a means to evaluate the effectiveness of the substitution matrices. The ability of amino acid substitution match to identify true homolog sequence matches were balanced against its ability to exclude false positives or unrelated sequences. Similar to previous studies [4, 14], coverage at 0.01 errors per query (CVE) was used as a means to evaluate the effectiveness of substitution matrices.

## Results and discussion

### Difference in the entropy level

RBLOSUM matrices identified as analogs to BLOSUM matrices are displayed in Table 1. For BLOCKS version 5, the entropy values are identical to those reported by Hess et al. and it is highly similar to verison13+. However, the entropy values differ largely for matrices computed using version 14.3.

**Table 1:** Matrices with respective entropy values (i) reported by Hess et al., (ii) present study

Matrix	(i) Hess et al.		(ii) Present study	
	Entropy	Bit units	Entropy	Bit units
BLOSUM50_5.0_	0.4808	1/3	0.4808	1/3
RBLOSUM52_5.0_	0.4918	1/3	0.4918	1/3
BLOSUM62 _5.0_	0.6979	1/2	0.6979	1/2
RBLOSUM64_5.0_	0.7003	1/2	0.7003	1/2
BLOSUM50_13+_	0.2430	1/4	0.1922	1/5
RBLOSUM59_13+_	0.2410	1/4	0.2411	1/4
BLOSUM62_13+_	0.3672	1/3	0.3173	1/3
RBLOSUM69_13+_	0.3601	1/3	0.3601	1/3
BLOSUM50_14.3_	0.1509	1/5	0.1198	1/6
RBLOSUM59_14.3_	0.1477	1/5	0.1537	1/5
BLOSUM62_14.3_	0.2685	1/4	0.2360	1/4
RBLOSUM69_14.3_	0.2662	1/4	0.2773	1/4

Previous studies have shown that the most appropriate way to compare two family of matrices is via entropy analogues. By comparing matrices with the same relative entropy, we can better assess the value or the correctness of the information encoded in the matrices [4, 20]. Therefore, different matrices can be compared and analyzed only if the difference of the entropy is relatively smaller between the matrices. Table 1 shows the similarities and differences of matrix entropies observed in the present study and reported by Hess et al.

BLOSUM50_14.3_ was compared with RBLOSUM59 (having entropies of 0.1509 and 0.1477 respectively) and BLOSUM62 was compared with RBLOSUM69 (having entropies of 0.2685 and 0.2662 respectively) reported by Hess et al. This comparison has revealed that the entropy of BLOSUM50_14.3_ is 0.1198 and the closest entropy observed is that of RBLOSUM56 (entropy of 0.1234). Similarly, the entropy for BLOSUM62_14.3_ is 0.2360 and we found RBLOSUM66 as the analog matrix (with entropy of 0.2445). Change in entropies clearly lead to different analog matrices. The RBLOSUM matrix variants discussed in this study and reported by Hess are shown in Additional file 3: Figure S4.

Hess et al. report that CorBLOSUM performs better in case of Astral data sets > 2.01. Changes in entropy lead to different analog matrices, with a few differences in the substitution score. In Additional file 4: Figure S5, shows the difference between RBLOSUM matrices of the present study and the matrix reported by Hess et al.

With the assumption that even slight variation in the matrix value may affect performance, authors have further done the performance analysis using pairwise sequence comparison and evaluation algorithm (PSCE) by Price et al. [14, 18].

### Evaluation of the matrices

**Fig. 1:**
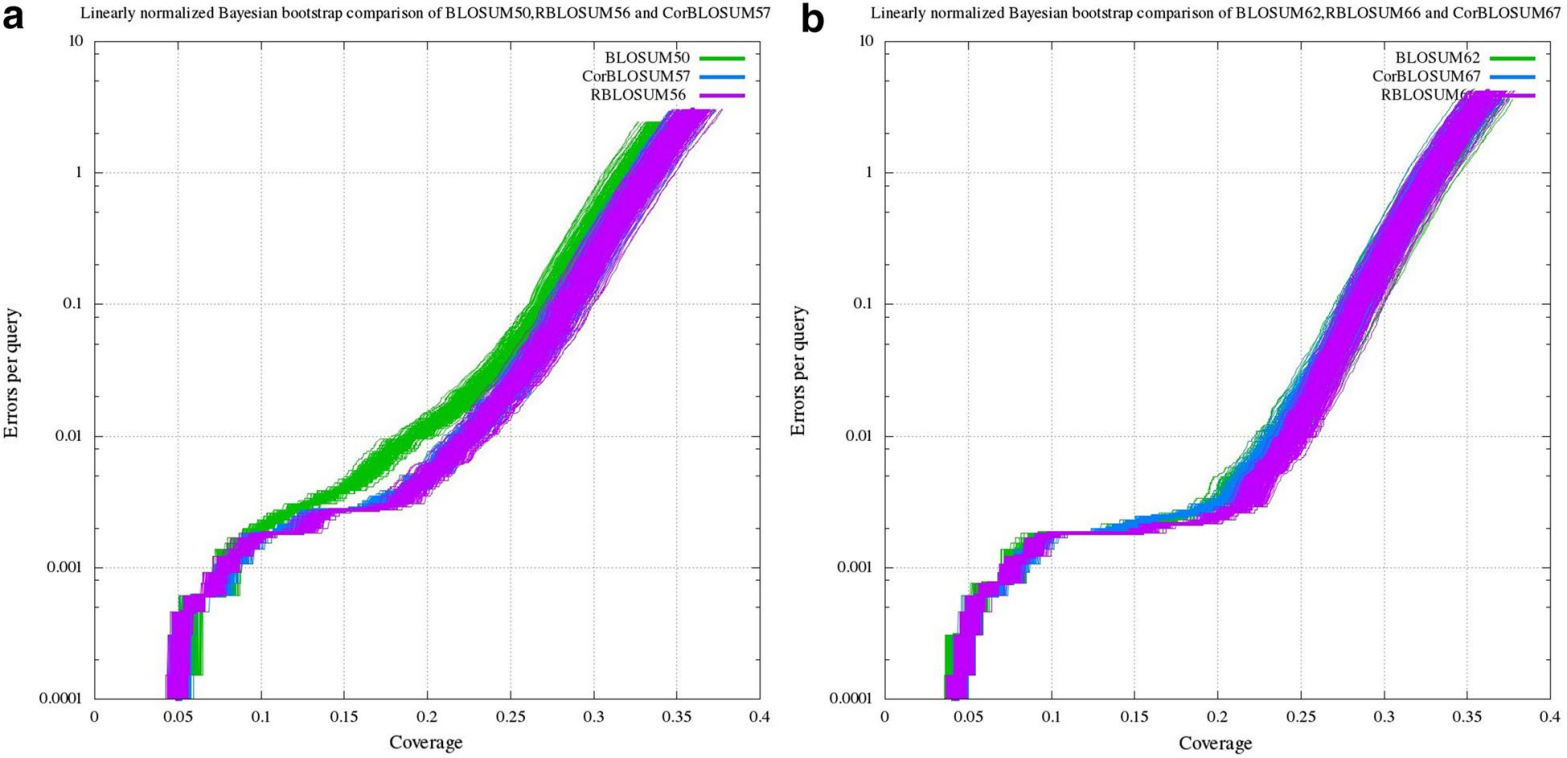
CVE plot showing the performance difference between the matrices for entropy level 50 and 62 using PSCE under linear normalization. **a** Performance difference between three matrix families for 50 entropy level using PSCE under linear normalization. **b** Performance difference between three matrix families for 62 entropy level using PSCE under linear normalization

Three different matrix families computed using BLOCKSv14.3 were compared and evaluated using different gap opening and extension parameters ranging from 11 to 16 and 1 to 2. The highest coverage obtained for different tested gap parameter are shown in Additional file 5: Table S1. The highest coverage was obtained for the gap opening and extension penalty of 12 and 1 respectively. Three matrix families were further evaluated on test database with the gap opening and extension penalty of 12 and 1 respectively, [21] using Bayesian bootstrapping to distinguish statistically. These search results were analyzed using CVE plots generated using PSCE tool. Figure 1 reports the CVE plot of different BLOSUM families. We reiterate the fact that even slight variation in matrix values can influence the performance [22] of RBLOSUM than CorBLOSUM. For instance, for entropy level 62, the graph in Fig. 1b clearly indicates that RBLOSUM66 finds more true homologs with minimum error per query than CorBLOSUM and BLOSUM matrices. In the case of entropy level 50 (see Fig. 1a), there is not much significant difference between the performance of RBLOSUM and CorBLOSUM matrices. Though the difference between the CVE lines are smaller, statistically they are significant (see Additional file 6). With quadratic normalization, we observed RBLOSUM66 as the best scoring matrix with a coverage of 0.451182, as shown in Table 2. Thus we argue that RBLOSUM outperforms the CorBLOSUM.

**Table 2:** Matrix family with coverage under quadratic normalization for the gap opening and extension penalty of 12 and 1 respectively

Matrix family	Matrix number	Coverage
BLOSUM	50	0.370823
RBLOSUM	56	0.418540
CorBLOSUM	57	0.413656
BLOSUM	62	0.436900
RBLOSUM	66	0.451182
CorBLOSUM	67	0.438116

In addition to the Astral v2.06, a few more Astral data set versions were also included in the analysis to reconfirm the performance of the matrices. RBLOSUM66 has been identified as the matrix with higher coverage and lesser error per query, from the inferred results of additional analysis (see Additional file 7).

## Conclusion and recommendation

In this paper, we have highlighted the entropy differences observed in RBLOSUM matrices as computed by us with that reported by Hess et al. The RBLOSUM matrices created in our study are substantially different from RBLOSUM of Hess et al. We have shown that it outperforms the CorBLOSUM and BLOSUM matrices. In a few cases tested by us, RBLOSUM and CorBLOSUM counterparts at the entropy level 50, performed almost equally with statistical significance. We emphasize that, with Astral dataset 2.06 and a few additional ASTRAL versions of dataset, RBLOSUM performs better than CorBLOSUM in terms of finding true homologous with less error per query. RBLOSUM performs statistically significant for the release of Blocks database version14.3. This difference in result reported by Hess et al. is due to selection of analog matrix with non-comparable entropy levels. We therefore refute the conclusion of Hess et al.

In a broader canvas, our work points to the need for reviewing the cardinal tools and techniques in science, taking into consideration the high quality data that is emerging with the advent of sophisticated instrumentation systems. Such investigations may reconfirm or correct existing tools and techniques.

## Limitation

The main limitation of the study is, different gap parameter tests were performed only on Astral v2.06 data set. Remaining additional data sets were evaluated using gap opening and extension penalty of 12 and 1 respectively, which was identified as the best gap parameter in the present study.

## Additional files

**Additional file 1.** Influence of increased blocks in computed matrices.

**Additional file 2.** Significance of improved matrices in similarity studies.

**Additional file 3: Figure S4.** Comparison of RBLOSUM matrices observed in the present study and Hess et al.

**Additional file 4: Figure S5.** Differences observed between RBLOSUM variants of present study and Hess et al.

**Additional file 5: Table S1.** Range of gap parameters evaluated using SSEARCH.

**Additional file 6.** Statistical significance of CVE lines between the matrices.

**Additional file 7.** Results and CVE plots of additional analysis performed on ASTRAL data sets.

## Abbreviations

PSCE: pairwise sequence comparison and evaluation tool
CVE: coverage vs error per query
