# Supplementary material for: RBLOSUM performs better than CorBLOSUM with lesser error per query
Source: BMC Res Notes. 2018 May 21;11:328. doi: 10.1186/s13104-018-3415-5 (PMC5963171; doi:10.1186/s13104-018-3415-5)
Supplement: Supplementary file 1 — Additional file 1. Influence of increased blocks in computed matrices. [file 13104_2018_3415_MOESM1_ESM.docx]

**Additional file 1 : Influence of increased blocks in computed matrices.**

Fig. S1 is an illustration of how amino acid substitution scores were varied between the BLOSUM62 in the year 1992 and 2017. The newly computed BLOSUM62 matrix from BLOCKSv14.3 is more diverse than BLOSUM625.0 developed using BLOCKSv5.0. This might be expected because of the increased protein sequences in the BLOCKS databases. As presented in Supplementary Fig. S1 and Fig. S2, diagonal elements of newly computed BLOSUM6214.3 matrix are greater than that of the BLOSUM625.0 matrix . Similarly off diagonal elements of the new BLOSUM6214.3 matrix are larger from 1 to 5 than those of BLOSUM625.0, although few off-diagonal elements are smaller. Notably, values for amino acid pair associated with Glutamic acid, Lysine, such as E—F, E—M and K—F, K—Y , K—V pairs are reduced in BLOSUM6214.3.


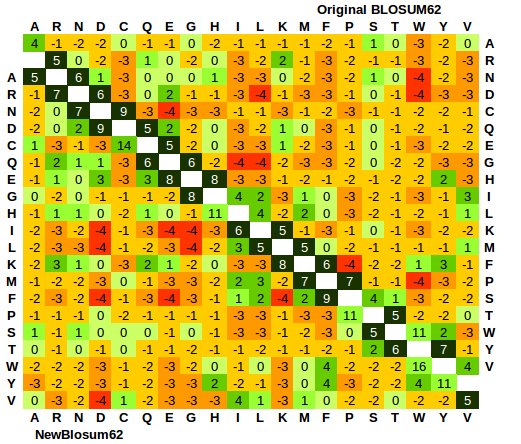


Fig. S1 Comparison between BLOSUM6214.3 and BLOSUM625.0. The obtained matrix (lower) and BLOSUM62 5.0 matrix (upper) are shown.


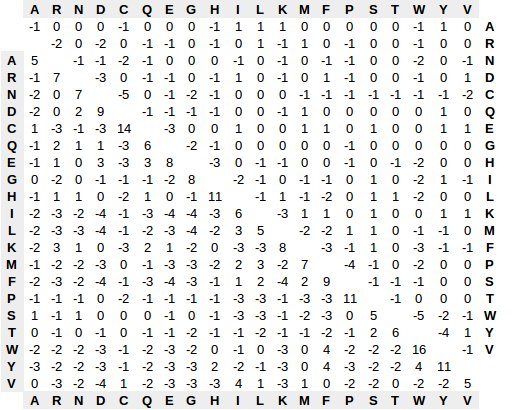


Fig. S2 BLOSUM6214.3 matrix and the difference observed between the matrices

The obtained matrix BLOSUM6214.3 (lower) and difference matrix (upper) obtained by subtracting BLOSUM625.0 from the obtained matrix are shown in Fig. S2. These differences in the substitution scores would influence the results of similarity studies. This recomputed matrices will provide a more sensitive substitution scheme for the better understanding of sequence similarity and evolutionary relationships. The study attempts to show the effects of updates to Blocks on the matrices derived from the database.
