# Supplementary material for: RBLOSUM performs better than CorBLOSUM with lesser error per query
Source: BMC Res Notes. 2018 May 21;11:328. doi: 10.1186/s13104-018-3415-5 (PMC5963171; doi:10.1186/s13104-018-3415-5)
Supplement: Supplementary file 2 — Additional file 2. Significance of improved matrices in similarity studies. [file 13104_2018_3415_MOESM2_ESM.docx]

**Additional file2: Significance of improved matrices in similarity studies.**

Development and improvement of the matrices are crucial for identifying and aligning more distant homologs with an increased accuracy in similarity studies. The improvement in the matrices tends to increase the statistical significance and accuracy of alignments. Many studies have been under- taken since the first compilation of such matrices and have been developed and improved[24]. Similarly, our analysis further justifies the fact that the slight variation in matrix can influence the performance as shown by Fig. 1 in the result and discussion session. The Fig. 1 compares the performance of RBLOSUM and CorBLOSUM matrices.

Fig.1 CVE plot showing the performance difference between the matrices for entropy level 50 and 62. a) Performance difference between three matrix families for 50 entropy level using PSCE under linear normalization b) Performance difference between three matrix families for 62 entropy level using PSCE under linear normalization**.
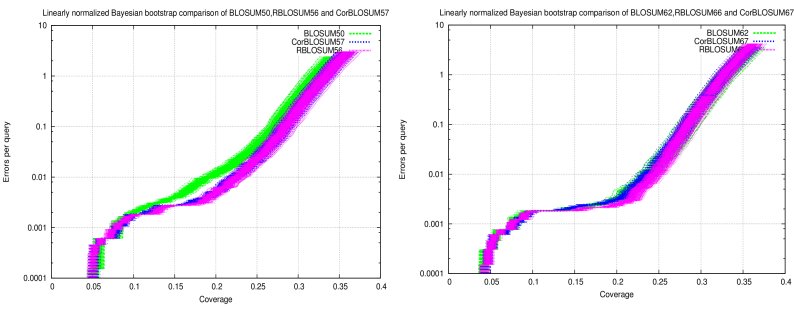
**

Fig. S3 Performance difference between BLOSUM62_5.0_ and BLOSUM62_14.3_ matrices using PSCE under linear normalization

Also analyzing the hits of BLOSUM62_5.0_ and BLOSUM62_14.3_, the newly computed BLOSUM contains 3151 unique hits which were not identified using BLOSUM_5.0._
