# Supplementary material for: RBLOSUM performs better than CorBLOSUM with lesser error per query
Source: BMC Res Notes. 2018 May 21;11:328. doi: 10.1186/s13104-018-3415-5 (PMC5963171; doi:10.1186/s13104-018-3415-5)

**Additional file 3:** Figure S4 Comparison of RBLOSUM matrices observed in the present study and Martin Hess *et al*

Fig. S4 Comparison of RBLOSUM matrices observed in the present study and Martin Hess *et al.* a)Matrix RBLOSUM66 below and RBLOSUM69_Martin_ above b) Matrix RBLOSUM56 below and RBLOSUM59_Martin_ above
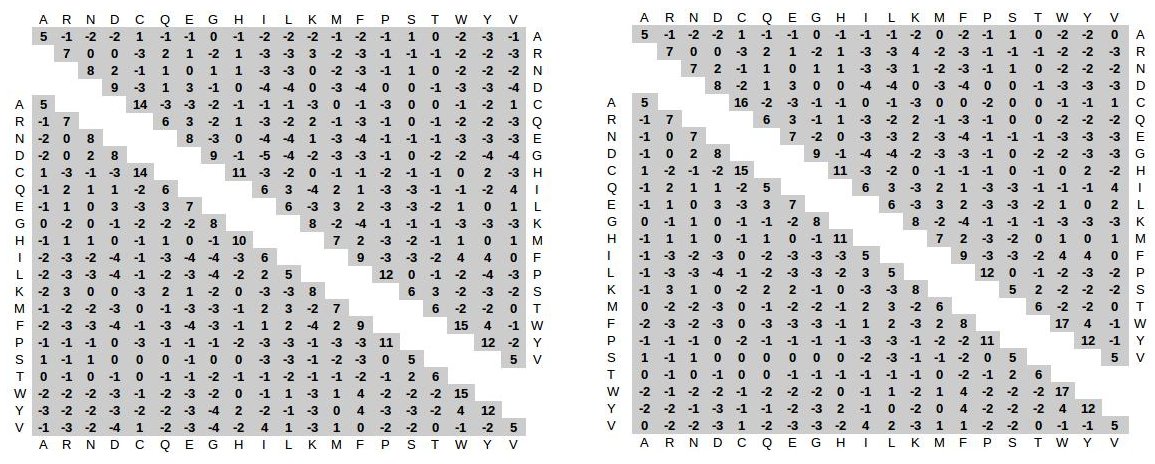

Supplement: Supplementary file 3 — Additional file 3: Figure S4. Comparison of RBLOSUM matrices observed in the present study and Hess et al. [file 13104_2018_3415_MOESM3_ESM.docx]
