# Supplementary material for: RBLOSUM performs better than CorBLOSUM with lesser error per query
Source: BMC Res Notes. 2018 May 21;11:328. doi: 10.1186/s13104-018-3415-5 (PMC5963171; doi:10.1186/s13104-018-3415-5)
Supplement: Supplementary file 4 — Additional file 4: Figure S5. Differences observed between RBLOSUM variants of present study and Hess et al. [file 13104_2018_3415_MOESM4_ESM.docx]

A**dditional file 4 :** Differences observed between RBLOSUM variants of present study and Martin Hess *et al*


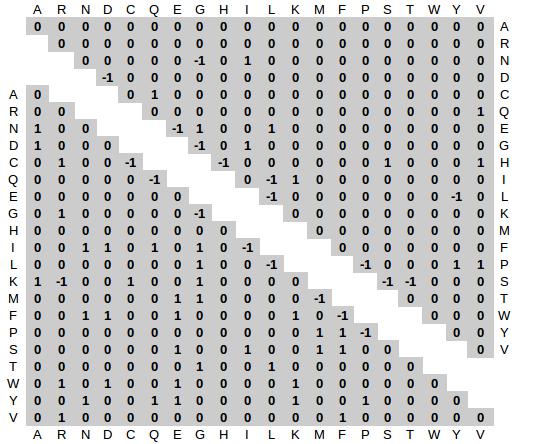


Fig. S5 Differences observed between RBLOSUM variants of present study and Martin Hess et al. Difference between RBLOSUM56 with RBLOSUM59_Martin_ and Difference between RBLOSUM66 with RBLOSUM69_Martin_ are displayed in the lower and upper diagonal respectively. The difference between the two substitution matrices values range from -1 to 1.
