# Supplementary material for: RBLOSUM performs better than CorBLOSUM with lesser error per query
Source: BMC Res Notes. 2018 May 21;11:328. doi: 10.1186/s13104-018-3415-5 (PMC5963171; doi:10.1186/s13104-018-3415-5)
Supplement: Supplementary file 5 — Additional file 5: Table S1. Range of gap parameters evaluated using SSEARCH. [file 13104_2018_3415_MOESM5_ESM.docx]

**Additional file 5: Table S1 Range of gap parameters evaluated using SSEARCH.**

| **Matrix family** | **Gap parameters** | **Coverage** | |
| --- | --- | --- | --- |
|  |  | **Original value** | **Round up value** |
| RBLOSUM66 | 11/1 | 0.239733 | 0.240 |
| CorBLOSUM67 | 11/2 | 0.254276 | 0.254 |
| RBLOSUM66 | 12/1 | 0.256649 | 0.257 |
| CorBLOSUM57 | 12/2 | 0.256149 | 0.256 |
| RBLOSUM66 | 13/1 | 0.249618 | 0.250 |
| CorBLOSUM67 | 13/2 | 0.256224 | 0.256 |
| CorBLOSUM67 | 14/1 | 0.252103 | 0.252 |
| RBLOSUM66 | 14/2 | 0.256457 | 0.256 |
| RBLOSUM66 | 15/1 | 0.254379 | 0.254 |
| RBLOSUM66 | 15/2 | 0.255581 | 0.256 |
| CorBLOSUM67 | 16/1 | 0.254849 | 0.255 |
| CorBLOSUM67 | 16/2 | 0.255946 | 0.255 |

Values reported in this table were identified as the highest coverage for each gap penalty parameters under linear normalization at 0.01 error per query.

Though the coverage values are quite similar, their performance differences are statistically significant with the improvement in remote homology detection compared with the classic BLOSUM and CorBLOSUM matrices. To determine which matrix performs better at a given error rate, matrices were ranked based on their coverage at a suitable error rate. The performance of each matrix family was judged by the coverage at 0.01 EPQ on a training data set, using different gap parameters that optimize the coverage. The obtained highest coverage value for each gap parameter are shown in Table S1. Using these matrices, there is a range around the optimum gap-parameter set in which the coverage does not vary widely. The best scoring matrix, with coverage of 25.7% (0.257), was RBLOSUM66 derived from BLOCKS 14.3 with gap open/extension parameters of 12/1 (Table S1).

CorBLOSUM67 (13/2) and RBLOSUM66 (14/2,15/2) scored equivalently at 25.6%. In other words the RBLOSUM66 matrices outperform the CorBLOSUM matrices by 1%. According to Green and Brenner these gains are significant, considering the maturity of Bayesian implemented PSCE technology [16]. “*Bayesian bootstrap ensemble does not exhibit a significant bias. Also notable is that the Bayesian coverage distributions are narrower*” [16].

200 bootstrap samples of each set of results were used to generate a coverage distribution at 0.01 error per query. The distribution can be used to determine the significance between coverage levels generated by any two matrices or parameter sets. We applied our analysis to the comparative study of BLOSUM matrix families and found that using RBLOSUM matrices results in a small, but statistically significant improvement in remote homology detection compared with the classic BLOSUM and CorBLOSUM matrices.
