# Supplementary material for: RBLOSUM performs better than CorBLOSUM with lesser error per query
Source: BMC Res Notes. 2018 May 21;11:328. doi: 10.1186/s13104-018-3415-5 (PMC5963171; doi:10.1186/s13104-018-3415-5)
Supplement: Supplementary file 6 — Additional file 6. Statistical significance of CVE lines between the matrices. [file 13104_2018_3415_MOESM6_ESM.docx]

**Additional file 6: Statistical significance of CVE lines between the matrices**

During the analysis using training data set, we identified the optimal gap parameters as (12, 1). The results were presented in CVE plots in Fig. S6 under linear normalization and it was identified that the differences between the coverage were not wide at an optimum range of 0.01 error per query [14].

Fig S6. *CVE plot showing the performance difference between the matrices for entropy level 62 using PSCE under linear normalization*

From the PSCE analysis it has been observed that RBLOSUM family of substitution matrices are most suitable for detecting remote homologues and recognizing a significantly larger fraction of relations than other matrices. Unique hits obtained from the PSCE analysis is reported in Table S2. The result clearly emphasize the fact that even though the difference between the coverage is small, there is a significant influence in identifying the remote homologues.

Table S2: The number of unique hits obtained for each matrix which was not contained in other matrices hits.

| **Compared matrices** | **Unique hits obtained** | | |
| --- | --- | --- | --- |
|  | **BLOSUM** | **RBLOSUM** | **CorBLOSUM** |
| RBLOSUM66vsCorBLOSUM67 |  | 2122 | 95 |
| BLOSUM62vsCorBLOSUM67 | 917 |  | 637 |
| BLOSUM62vsRBLOSUM66 | 227 | 1974 |  |
|  | | | |
| RBLOSUM56vsCorBLOSUM67 |  | 1430 | 409 |
| BLOSUM50vsCorBLOSUM57 | 12 |  | 5984 |
| BLOSUM50vsRBLOSUM62 | 14 | 7007 |  |

To determine the significance of the coverage differences generated by each substitution matrix, we did bootstrap analysis on each. 200 bootstrap samples of each set of results were used to generate a coverage distribution at 0.01 error per query as shown in Fig. S7.

Fig. S7 *CVE plot with bootstrap replicates showing the performance difference between the matrices using PSCE under linear normalization
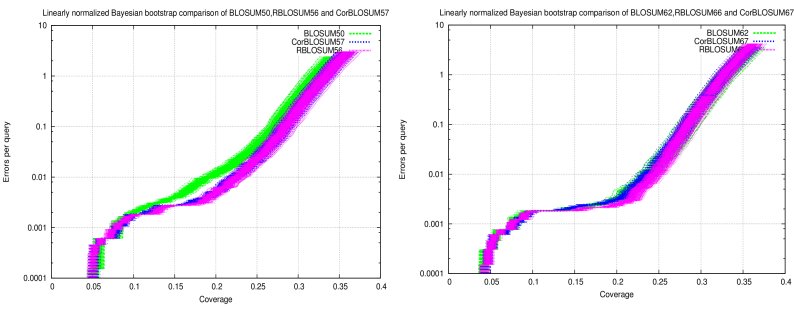
*

From Fig. S7, it can be interpreted that the widths of these distributions are large compared with the average differences in coverage. This is because of the number of bootstrap replicate distribution for each family at 0.01 error per query. These overlapping distributions make it difficult to distinguish the performance of BLOSUM, RBLOSUM and CorBLOSUM matrices. However, the statistic of interest in this analysis suggested by PSCE, is not the difference in mean coverage, rather it is the mean difference in coverage [16]. This distinction is significant, since the results obtained from a single data replica are correlated across different parameters [16].

Statistical significance between the coverage distributions can be further tested using the 95% confidence interval for each pair of families. If the confidence interval value includes 0, it indicates that the fact the performance of both substitution matrices are same at 95% confidence. The confidence interval for each pair of matrices are listed in Table S3.

Table S3 Confidence Interval observed between the tested matrices

| **Matrix pairs** | **Confidence Interval** |
| --- | --- |
| BLOSUM50 vs RBLOSUM56 | [0.0248 , 0.042] |
| RBLOSUM56 vs CorBLOSUM57 | [-0.006 , 0.0201] |
| BLOSUM62 vs RBLOSUM66 | [0.0212 , 0.0710] |
| RBLOSUM66 vs CorBLOSUM67 | [0.0075 , 0.0208] |

From the Table S3 it has been observed that none of the 95% confidence intervals generated by the matrices includes the value 0 in the confidence interval except RBLOSUM56 vs CorBLOSUM57. RBLOSUM56 and CorBLOSUM57 performs similarly while others are different as discussed in the conclusion. Thus, after extensively studying these three families, RBLOSUM matrices show a small, statistically significant improvement in remote homology detection compared with the classic BLOSUM and CorBLOSUM.
