# Supplementary material for: RBLOSUM performs better than CorBLOSUM with lesser error per query
Source: BMC Res Notes. 2018 May 21;11:328. doi: 10.1186/s13104-018-3415-5 (PMC5963171; doi:10.1186/s13104-018-3415-5)
Supplement: Supplementary file 7 — Additional file 7. Results and CVE plots of additional analysis performed on ASTRAL data sets. [file 13104_2018_3415_MOESM7_ESM.docx]

Additional file 7: Comparison of three matrix families, for the different Astral data set version for the entropy level 50 and 62 under linear normalization.

Fig. S8 CVE plot of three matrix families for the Astral version 1.75
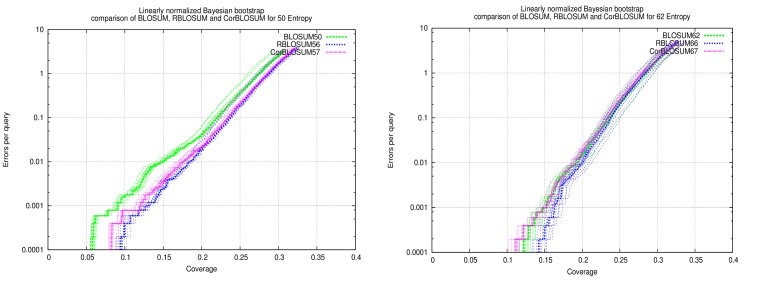


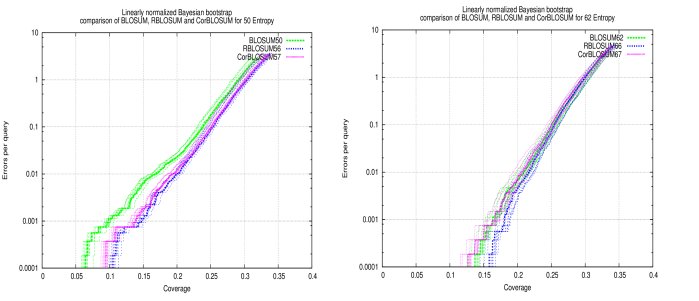


Fig. S9 CVE plot of three matrix families for the Astral version V2.01


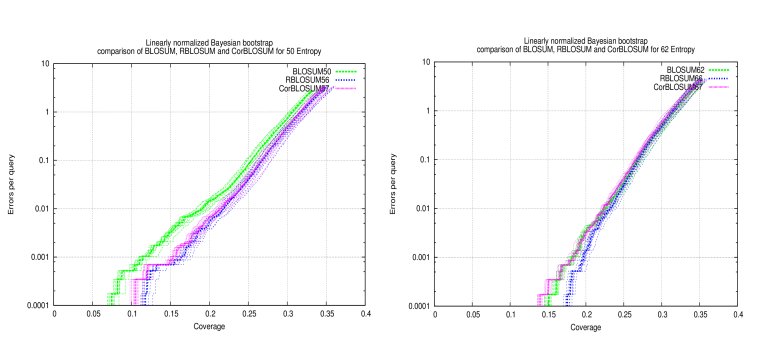


Fig. S10 CVE plot of three matrix families for the Astral version V2.03


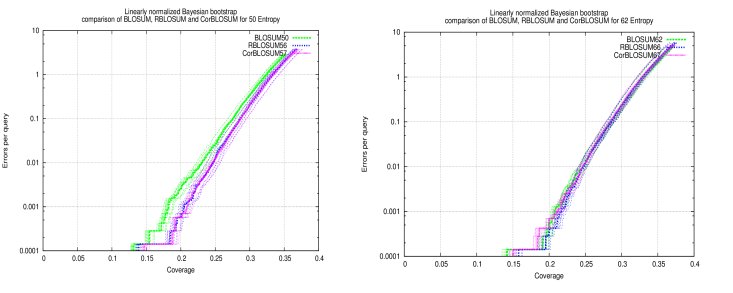


Fig. S11 CVE plot of three matrix families for the Astral version V2.05

Further more additional analysis were performed on data set Astral v1.7, 2.1, 2.3, 2.5. Astral version 1.7 is the updated hand curated dataset and the remaining are the most recently updated and automated alternative versions. Similarity search was performed on these ASTRAL data sets using the three different matrices and their performance was evaluated using PSCE tool. RBLOSUM matrices perform better with higher coverage for the ASTRAL data sets version 1.75, 2.01 and 2.03. In the case of ASTRAL data set version 2.05, RBLOSUM and CorBLOSUM CVE plots in Fig S11 overlap each other and performs equally with no significant difference.
